# A Case of Incapsulated Pleuritic Effusion

**Published:** 1873-02

**Authors:** John E. Lockridge

**Affiliations:** Augusta County, Virginia


### SOUTHERN MEDICAL RECORD.

VOL. III.—FEBRUARY, 1873.—NO. 2.

### PART I.

### ORIGINAL COMMUNICATIONS.

### A CASE OF INCAPSULATED PLEURITIC EFFUSION.

BY JOHN E. LOCKRIDGE, M.D., OF AUGUSTA COUNTY, VIRGINIA.

On November 11, 1870, I was called to see J. W. H., aged seven years. I found him suffering from quite a sharp attack of pleuro-pneumonia; both were in the first stage, and confined to about the lower half of the upper lobe of the left lung. His previous health had been rather unsatisfactory; I had prescribed for him a few days before for an attack of asthma. For the past six months or more his color had been very unhealthy-looking, and his appetite capricious, during which time he had scarcely partaken of any animal diet; and whilst he had not been particularly sick, yet he was languid, and his color very sallow in appearance—presenting the so-called "bilious" appearance.

I found him with considerable fever, pain in his side, cough, etc. Over the affected zone the friction sound and crepitant rale were heard. I put him on a mixture of camph. tinct. opii., antimonii vini, and sp. nitric ether, and ordered mustard plaster to affected side. The pneumonia very soon ran into the second stage, and in a day or two I put him on Dover's powder and quinine, with a blister to his side; also, some brandy, and soup, milk, etc. Under this treatment he gradually improved, so that on the 21st of November the symptoms had nearly all subsided; there were remaining very slight dullness on percussion, and very faint bronchial respiration and broncophony over a very small portion of the affected side; there was no fever, his respiration was nearly natural, his appetite returning, and, in short, the case seemed to be doing so well, that I lost sight of him until the 30th, when I was recalled to find my patient in a very bad condition.

November 30.—I found my patient propped up in bed, and breathing fifty-six times in a minute; his heart was beating tumultuously, one hundred and seventy times to the minute, beneath the *right nipple;* pulse at the wrist feeble; complaining of pain about the chest; percussion, to my surprise, revealed a dull zone corresponding to about the *lower half only* of the upper lobe of the left lung; on ascultation, bronchial breathing and broncophony corresponded to the same zone; the entire lower lobe and the upper part of the upper lobe yielded a perfectly clear sound on percussion, with the healthy respiratory murmur; there was no ægophony over diseased part. Inspection and measurement yielded negative results; the sides of the chest measuring precisely the same, and there appearing to be no bulging of the intercostal spaces. Nor could I learn anything from position, the same sounds being elicited in every position in which I placed my patient. By means of exclusion I arrived at the diagnosis of incapsulated pleuritic effusion.

I prescribed iodide of potassium in five-grain doses *ter die;* ordered his side to be painted with tincture of iodine every six hours, first over a space of about four by four inches in front until the part became too much vesicated, then select a portion of the surface over lower part of scapula of like extent, and alternate before and behind as the parts became too sore. For the terrible cough, and as somewhat of a diuretic, I prescribed the following:

℞.—Tinct. opii. camph., sweet spts. nitre, aa ℥ ss; acidi hydrocyanic dil., (U. S. P.) ʒ i; syrup simplex, sol. gum Arabic, aa ℥ iss. M. S.—Take a teaspoonful every three or four hours, or *pro re nata.* Ordered a diet of soup, milk, etc.

The above treatment was continued for several weeks, during which time I had the satisfaction of seeing the heart gradually return from its remarkable pilgrimage towards the right border of the sternum; but at about three weeks from the commencement of the treatment for the effusion, I discovered that tubercles were forming beneath the left clavicle. There was, first, very slight rough breathing, which was soon followed by slight dullness, and bronchial respiration and broncophony; and this deposition of tubercles was separate from the dullness from the effusion. In place of the iodide of potassium, syrup of the iodide of iron and cod-liver oil were substituted, sometimes separately and sometimes combined. The diuretico-expectorant mixture was continued, which was strengthened by the addition of grs. ii. sul. morphiæ to each four ounces of the mixture. The tinct. iodine was continued locally all the time; and a liberal allowance of brandy was now given daily, and as generous a diet as the patient could be induced to take was allowed.

This treatment was continued, without interruption, throughout the months of December, January, February and March, the symptoms gradually but very slowly subsiding. The heart reached the right border of the sternum, then the middle, then the left border, and finally it resumed its position amongst the great organs of the body. The dullness gradually diminished in area, both from the effusion and the deposition of tubercles; the bronchial breathing gradually yielded to the welcome respiratory murmur; the pulse and respiration diminished in number to the minute; the patient's comfort gradually increased, and disease was gradually being dethroned, and health was assuming its supremacy in the castle. But, in the meantime, gallon after gallon of brandy and whisky—and alcohol, which was as efficacious and cheaper than either—were being consumed; bottle after bottle of cod-liver oil, and ounce after ounce of syrup of iodide of iron and tincture of iodine were being consumed, until the 31st of March I dismissed the patient cured—there being no sign of a tubercle in the lungs, or of effusion of serum in the pleural cavity. I have often regretted that I kept no account of the amount of liquor consumed by this little boy; but the quantity of spirits and cod-liver oil was immense, and I fear that, if I were able to report the exact amount, my readers would regard it as almost fabulous. Suffice it to say, that the little fellow soon became interested, as it were, in the treatment of his own case, and when the time would come for him to take his remedies, he would clamor for them, and just as soon as he became strong enough to walk across the room, he would help himself to his medicine in case he were not promptly served by his attendants.

About one year ago this boy passed through a very severe attack of pertussis without even the necessity of calling in a physician to his aid. About two months ago (October 6, 1872) his father brought him to my office and desired me to prescribe for him for catarrhal jaundice, and I took the opportunity to examine his lungs and heart carefully, and found everything to be in sound and perfect order. One week later I saw him, and his jaundice was entirely relieved, and he presented as perfect a picture of health as I have ever seen.

To my mind, this case presents several points of very great interest. In the first place, the diagnosis was difficult to make, and, without a preceding knowledge of the history, it would have been next to impossible. The position of the heart so exceedingly far to the right; the circumscribed dullness, bronchial breathing and broncophony; together with the healthy respiratory murmur both above and below, and the absolutely negative evidence afforded by inspection, position and measurement, gave rise, at first, in my mind, to the opinion that there existed some kind of tumor—either cancer of the lung or mediastinal tumor. But this notion soon vanished when I reflected that just nine days before, at my last visit, the heart was beating in its normal position, and that there existed at that time a very slight degree of dullness on percussion; and that, I now believe, was incipient effusion of serum, instead of the remains of the late pneumonic block. These facts seemed to show clearly that nothing could have so quickly displaced an organ save an accumulation of serum, pus or blood. And on the other hand, it seemed to me to be next to impossible that such a water-tight adhesion of the pleural surfaces could exist as to withstand so great a pressure and displacement of organs, and not even allow an appreciable amount of serum to gravitate to the base of the thorax. I mentioned this case to one of my accomplished medical friends, and he was very plainly of the opinion that such an adhesion was impossible. But such a conclusion I arrived at, and directed my treatment accordingly, with the most happy results.

It seems that the thrusting of the heart to the right of the sternum by effusion into the pleural cavity is rather a rare occurrence, for Sir Thomas Watson says that he has not seen more than a dozen cases, and that Andral had only seen one well-marked case. Incapsulated effusion is doubtless still more uncommon, inasmuch as all of the authorities on the theory and practice of medicine at my command speak of it in a vague manner, as something that has, or might occur, without giving any precise rules for its diagnosis. They all seem to speak of such an encysted accumulation as being very small in amount, and scarcely at all recognizable. The only thing of a positive character that led me to a correct conclusion was the very sharp pleurisy that existed two or three weeks before I discovered the great effusion; everything else was of a negative character.

Another feature of importance in this case is the readiness with which this long-compressed portion of the lung returned to its healthy condition. It was subjected to very great pressure for at least a period of three months—four months having elapsed from the time of effusion to that of complete absorption. Yet, as the absorption progressed, the lung at once seemed to be in a condition to perform perfectly its function. After complete absorption, we would naturally have looked for some sinking in of the chest, incident to a lung diminished in size by adhesions, or a condition approaching to carnification from long compression; but nothing of the kind occurred in the slightest degree. This, however, may be accounted for from the fact that, although the compressing force must have been very considerable, yet at no time was the lung reduced to that condition generally known as carnification; for, at all stages of the treatment, bronchial breathing was plainly audible at the point of compression, and the healthy respiratory murmur existed beyond that point—thus showing that air passed and repassed freely the point of effusion. This is doubtless a prognostic point of some importance in the treatment of these rare cases of incapsulated pleuritic effusion, in reference to the matter of paracentesis thoracis, or the safety and propriety of effecting absorption of the fluid by the slower means of remedial agents. In case the effusion is great, as in the case under consideration, but not postively threatening life; and provided we are perfectly satisfied, as in this case, that air passes and repasses the point of compression, we are justified in waiting the action of remedies, without running any very serious risk of having a lung, or a portion of a lung, impervious to air in the future, and a chest sunken in, in consequence.

This naturally brings us to the consideration of the propriety of thoracentesis on the one hand, or to trusting to the absorptive powers of certain remedies on the other hand, in these or any other cases of pleuritic effusion. I notice in the *London Lancet* of March last a discussion in the Clinical Society of London, in which Drs. Murray, Moxon, Anstie, Playfair, Wilks, and others, participated, for and against thoracentesis, and I was very much surprised to find that the operation was recommended by some of those distinguished men in almost all cases of effusion; and that, too, apparently without resorting to—or, at least, without spending much time in waiting for—the action of remedies. If the patient's life be in very great jeopardy, or if the most potent remedies have been tried long and tried in vain; and if the lung, or even a portion of it, has been subjected to very great pressure for a long time; or we have every reason to believe that the lung is reduced to a state of carnification, and there be a fear that, without immediate relief, it will be reduced to a condition of worthlessness; then, of course, the operation should be performed without delay. But to resort to the operation, with its dangers from the admission of air, just because it is a speedy mode of giving relief, perhaps only temporary, seems to me to be reprehensible. And this is especially the case when I reflect that I have never had to resort to the operation in any case, although I have treated a goodly number of cases of pleuritic effusion, and have seen cases in the practice of other physicians, and nearly every one recovered by the use of remedies. I have no recollection of but one death from effusion in my practice; and I am quite sure thoracentesis would not have saved this very old woman.

I cannot close this paper without bearing testimony to the wonderful effect of the preparations of iodine in the treatment of these effusions, used both internally and externally. There is nothing in my mind surer, than that tincture of iodine, painted over the chest walls, and carried to the point of vesication, will sooner or later effect the absorption of effused serum; or hasten the resolution of a hepatized lung; or promote the absorption of the so-called cheesy or inflammatory tuberculosis, especially when combined with syrup of iodide of iron or cod-liver oil. And, as a general thing, the action, or rather their action, is sufficiently and satisfactorily speedy in the cases in question.

The only apology I have for reporting this case is, that I hope it may aid some poor fellow in making his diagnosis in a similar one, for I am sure that I have not been so perplexed for a number of years to make a diagnosis in any case of thoracic disease as I was in this one. My young friend, Dr. J. W. Harris, visited the case once with me, and looked upon the position of the heart as being most remarkable, and the nature of the case as being entirely different from anything that he had seen, or that had been discussed in lectures. The Doctor, who is present at this writing, reminds me that, at the time he saw the case with me, I caused the boy to stand up on his feet for ten minutes or more, to test whether the serum would gravitate to the base of the chest through the adhesion; but the clearness on percussion persisted.

*November* 30, 1872.

